# *Drosophila melanogaster* Oocytes after Space Flight: The Early Period of Adaptation to the Force of Gravity

**DOI:** 10.3390/cells11233871

**Published:** 2022-12-01

**Authors:** Irina V. Ogneva, Maria A. Golubkova, Nikolay S. Biryukov, Oleg V. Kotov

**Affiliations:** Cell Biophysics Laboratory, State Scientific Center of the Russian Federation Institute of Biomedical Problems of the Russian Academy of Sciences, 76a, Khoroshevskoyoe Shosse, 123007 Moscow, Russia

**Keywords:** cellular mechanoreception, space flight, oocyte, cytoskeleton, cell stiffness

## Abstract

The effect of space flight factors and the subsequent adaptation to the Earth’s gravity on oocytes is still poorly understood. Studies of mammalian oocytes in space present significant technical difficulties; therefore, the fruit fly *Drosophila melanogaster* is a convenient test subject. In this study, we analyzed the structure of the oocytes of the fruit fly *Drosophila melanogaster*, the maturation of which took place under space flight conditions (the “Cytomehanarium” experiment on the Russian Segment of the ISS during the ISS-67 expedition). The collection of the oocytes began immediately after landing and continued for 12 h. The flies were then transferred onto fresh agar plates and oocyte collection continued for the subsequent 12 h. The stiffness of oocytes was determined by atomic force microscopy and the content of the cytoskeletal proteins by Western blotting. The results demonstrated a significant decrease in the stiffness of oocytes in the flight group compared to the control (26.5 ± 1.1 pN/nm vs. 31.0 ± 1.8 pN/nm) against the background of a decrease in the content of some cytoskeletal proteins involved in the formation of microtubules and microfilaments. This pattern of oocyte structure leads to the disruption of cytokinesis during the cleavage of early embryos.

## 1. Introduction

Maintaining the human reproductive system is one of the conditions for deep space exploration. The female reproductive system deserves special attention due to its role not only in the healthy aging of women who have been in space for a long time, but also in the evolutionary aspect of maintaining the species. However, to date, there is a very limited number of studies on female reproductive health after exposure to space flight factors [1,2] and there are practically no data on the structure of oocytes after such exposures. This is due to the large number of difficulties in organizing such experiments involving mammals in space or immediately after returning to Earth.

A significantly more convenient test subject, from the point of view of organizing a space experiment, is the fruit fly *Drosophila melanogaster*. On the one hand, the maturation of fly and mammalian oocytes differs in a number of fundamental ways, but on the other hand, the structures of the mature oocytes have much in common. Thus, the mitochondria necessary for the energy supply of the future embryo are provided by the oocyte, as well as the cytoskeletal structures, for the formation of the spindle and the contractile ring, which are necessary at the early stages of embryo cleavage.

The results of a few simulation experiments indicate that cultivation under conditions of simulated microgravity leads to the vacuolarization of the mitochondria and cytoplasm, and the disruption of the structure of microvilli in the zona pellucida of mouse preantral follicles [3]. In mouse oocytes cultured in simulated weightlessness for up to 16 hours, abnormal spindle formation took place, probably as a result of gamma-tubulin redistribution [4]. Similarly, under the conditions of simulated microgravity in the ovaries of flies, there are also changes in cellular respiration (mediated by mitochondria) and various changes in the structure of the cytoskeleton [5,6]. Moreover, changes in the functional status of mitochondria can also be mediated by the cytoskeleton [7].

In general, the change in the structure of the cytoskeleton of various cell types, regardless of the degree of differentiation, under conditions of real or simulated weightlessness, as well as under hypergravity conditions, is a well-documented fact [8]. The violation of the structure of microtubules, intermediate filaments, and microfilaments is noted in many studies [9,10,11,12,13,14,15,16,17]. However, such changes in oocytes can have fatal consequences in early embryogenesis.

In previous studies, three consecutive generations of *Drosophila* were obtained in space flight, then the fourth generation, on Earth, and then the fifth generation, again in space [18]. However, we did not evaluate the fecundity of flies either in space flight or on Earth. At the same time, a decrease in the speed of movement of spermatozoa and a violation of the structure of their cytoskeleton in the early period of readaptation to gravity after a space flight [19] require both an analysis of the structure of the female germ cell and, in general, an assessment of the reproductive potential. 

All of the above suggests that oocytes that matured under space flight conditions may be particularly sensitive to the effects of overloads during landing and the period of adaptation to Earth’s gravity. Therefore, the aim of the study was to evaluate the structural changes in the oocytes of the fruit fly *Drosophila melanogaster*, which were collected immediately after the space flight. Due to the limited number of cells obtained, the cytoskeleton structure was assessed by determining the integrative parameter—cell stiffness—by atomic force microscopy. We found a decrease in the stiffness of the cortical cytoskeleton and the content of cytoskeletal proteins, which may have a negative effect on the early development of the embryo.

## 2. Materials and Methods

### 2.1. Experimental Design

The “Cytomehanarium” space experiment, as part of a real space flight aboard the RS ISS, was carried out from 21 to 29 September 2022 (ISS-67 Expedition).

In this experiment, 5 days before the launch of the space flight, 50 female larvae of the 3rd age of the fruit fly *Drosophila melanogaster* (Canton S line) were placed in 50 mL Falcon-type tubes with a breathable cap: 4 tubes for the flight group (group F) and 4 tubes for the synchronous control group on Earth (group S). Each Falcon contained a standard medium for breeding *Drosophila* with the following composition: water—1000 mL, agar-agar—7 g, granulated sugar—40 g, semolina—40 g, baker’s yeast—25 g, propionic acid—10 mL. 

The flies hatched at the end of the first day on board the ISS. The temperature regime of the synchronous control group S was observed according to the temperature graph of the space flight group F.

At the landing place (L), the flies from the flight group and the synchronous control group were transferred into a cage with agar plates for oocyte collection. Agar plates were prepared on the morning of the landing day in accordance with [20].

Within 12 h after landing (period L—L + 12 h), the cages with flies were transported to the laboratory at a temperature of + 25 °C. After arrival at the laboratory, the flies were transferred into cages with fresh agar plates and oocytes continued to be collected for the next 12 h (period L + 12 h—L + 24 h). 

The oocytes of the flight group F and the synchronous control group S obtained during the period L—L + 12 h were dechorionized with sodium hypochlorite (as a 50% bleach) according to the protocol in [20]. Then the dechorionized oocytes were transferred to the substrate and the stiffness was measured by atomic force microscopy. The limited number of oocytes only allowed for the measurement of stiffness in this group.

The oocytes obtained in the period L + 12 h—L + 24 h were also dechorionized as described above. Part of the oocytes was used to determine the stiffness. The rest of the oocytes were randomly divided into three biological replicates and frozen for subsequent protein isolation.

All the experimental procedures were approved by the Commission on Biomedical Ethics of the Institute of Biomedical Problems (IBMP), the State Scientific Center of the Russian Federation and the Federal State Budgetary Institution of Science (Minutes No. 521 dated 25 September 2019).

### 2.2. Atomic Force Microscopy

The stiffness of the oocytes was measured using an NTEGRA NEXT II atomic force microscope (NT-MDT, Moscow, Russia) using the technique described by us earlier [21,22] in the following modification. 

The stiffness was measured using a soft silicon cantilever (#CS17, NT-MDT, Moscow, Russia) with a spring constant range of 0.06–0.4 N/m. In the semi-contact mode, the resonant oscillation frequency of the cantilever was determined, and its spring constant kc was calculated, which in this work was 81 pN/nm.

The dechorionized oocytes were placed on a rigid substrate in a well for measurements using an atomic force microscope. The stiffness was measured in the contact mode. First, the calibration force–distance curves were obtained on a substrate outside of the oocytes. Furthermore, similar curves were obtained by indenting oocytes to a depth of 50 nm. The actual indentation depth and applied force were calculated using the following formula: h_s_ = x − y · a, F_s_ = y · a · k_c_, where hs is the actual indentation depth (m), F_s_ is the actual force applied to a cell (N), and k_c_ is the cantilever stiffness coefficient. The change in applied force was determined and the cell stiffness was estimated at an indentation depth of 50 nm using the following formula: k_s_ = F_s_/h_s_.

### 2.3. Western-Blotting

Total protein was isolated from the frozen dechorionized oocytes in Laemmli buffer with the addition of proteinase inhibitors (Calbiochem, San Diego, CA, USA). The protein concentration in each sample was measured and, accordingly, the same amount of protein was applied to the wells of the polyacrylamide gel. Denaturing gel electrophoresis and subsequent immunoblotting were carried out on a nitrocellulose membrane according to the standard procedure. We used primary antibodies to beta-actin (Act, #ab227387, Abcam, Cambridge, UK), singed (Sn, sn 7C was deposited to the DSHB by Cooley, L., DSHB Hybridoma Product, Iowa City, IA, USA), spectrin alpha (AlphaSpec, 3A9 was deposited to the DSHB by Branton, D./Dubreuil, R., DSHB Hybridoma Product, Iowa City, IA, USA), alpha-actinin (Actn, #ab50599, Abcam, Cambridge, UK), alpha-tubulin (AlphaTub, #ab52866, Abcam, Cambridge, UK), acetylated alpha-tubulin (ac-AlphaTub, #sc-23950, Santa Cruz Biotechnology, Inc., Santa Cruz, CA, USA), and laminB (LamB, ADL40 was deposited to the DSHB by Fisher, P. A., DSHB Hybridoma Product, Iowa City, IA, USA) at the manufacturers’ recommended dilutions, and their corresponding HRP-conjugated secondary antibodies (anti-rabbit #7074S, anti-mouse #7076S, all Cell Signaling Technology, Danvers, MA, USA). Next, membranes were treated with substrates (SuperSignal™ West Femto Maximum Sensitivity Substrate, Thermo Scientific, Waltham, MA, USA), detected using the ChemiDoc XRS+ imaging system (Bio-Rad Laboratories, Hercules, CA, USA), and processed using Image Lab Software v. 5.0 (Bio-Rad Laboratories, Hercules, CA, USA).

### 2.4. Statistical Analysis

When measuring stiffness, each oocyte was tested at least 5 times, and at least 30 oocytes were tested in each experimental group. When analyzing the content of proteins, at least three biological replicas were used for each group.

The results were analyzed using two-way ANOVA, using Student’s posterior *t*-test with Bonferroni’s correction for multiple comparisons. A significance level of *p* < 0.05 was used to assess the significance of changes. Data are presented as M ± SE (M is the mean, SE is the standard error of the mean).

## 3. Results

### 3.1. Stiffness of the Oocytes

**Figure 1:**
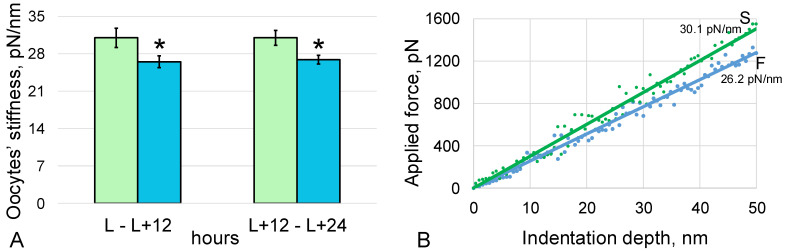
*Drosophila melanogaster* oocyte stiffness measured by atomic force microscopy (**A**), with typical force–distance curves (**B**). S—oocytes from the synchronous control group (green color), F—oocytes from the space flight group (blue color). *—*p* < 0.05 in comparison with the synchronous control group S.

The stiffness of oocytes laid by virgin *Drosophila melanogaster* females of flight group F during the first 12 h after landing of the spacecraft (period L—L + 12 h) was 26.5 ± 1.1 pN/nm and was 15% (*p* < 0.05) lower compared to the synchronous control group S, where the oocyte stiffness was 31.0 ± 1.8 pN/nm (Figure 1).

The stiffness of oocytes laid in the next 12 h (in the period L + 12 h—L + 24 h) was the same in both groups: in group F it was 26.9 ± 0.8 pN/nm, which was 13% (*p* < 0.05) lower than in the synchronous control group S, 31.0 ± 1.4 pN/nm.

### 3.2. Relative Content of the Cytoskeletal Proteins

In the first 12 h after landing (period L—L + 12 h), a small number of oocytes were obtained and used for stiffness measurements. In the following 12 h (between L + 12 h and L + 24 h), enough oocytes were obtained to measure stiffness, and three biological replicas were obtained to determine the relative content of cytoskeletal proteins.

**Figure 2:**
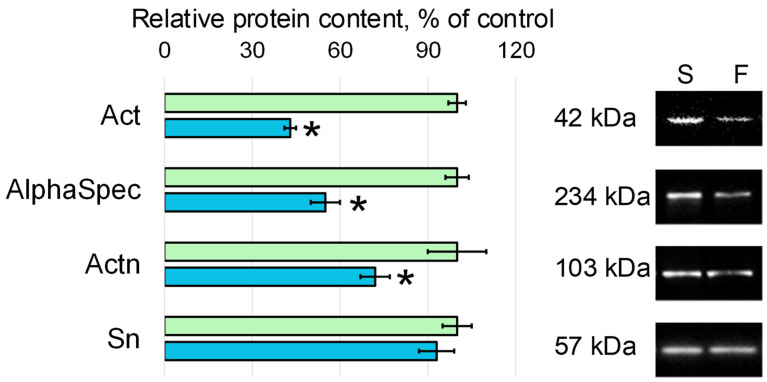
Relative content of proteins—components of microfilaments with typical Western blots. Act—beta-actin, AlphaSpec—spectrin alpha, Actn—alpha-actinin, Sn—singed. S—oocytes from the synchronous control group (green color), F—oocytes from the space flight group (blue color). *—*p* < 0.05 in comparison with the synchronous control group S.

In the flight group, the relative content of beta-actin (Act) was lower than in the synchronous control group by 57% (*p* < 0.05), and actin-binding proteins, spectrin alpha (AlphaSpec) and alpha-actinin (Actn), were lower by 45% (*p* < 0.05) and 28% (*p* < 0.05), respectively (Figure 2). The content of another actin-binding protein, singed (Sn), in the flight group did not differ from the level found in the control group.

**Figure 3:**
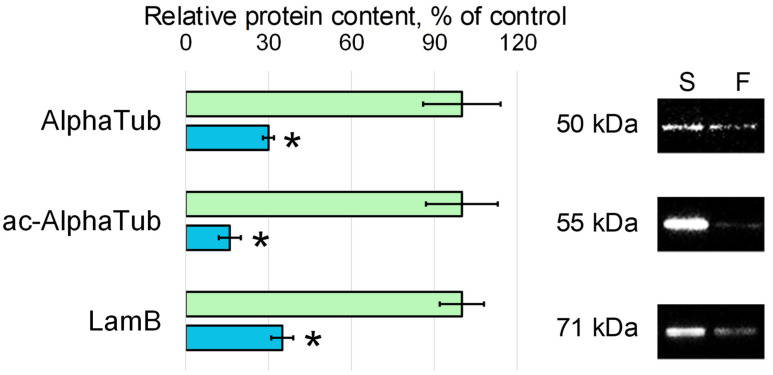
Relative content of alpha-tubulin (AlphaTub), acetylated alpha-tubulin (ac-AlphaTub), and Lamin B (LamB) with typical Western blots. S—oocytes from the synchronous control group (green color), F—oocytes from the space flight group (blue color). *—*p* < 0.05 in comparison with the synchronous control group S.

The decrease in the content of microtubule components and intermediate filaments in group F oocytes compared to the synchronous control group S was more dramatic; the relative content of alpha-tubulin (AlphaTub) in group F decreased by 70% (*p* < 0.05), acetylated alpha-tubulin (ac-AlphaTub) by 84% (*p* < 0.05), and lamin B (LamB) by 65% (*p* < 0.05) (Figure 3).

## 4. Discussion

The study of cells on Earth, the maturation of which took place under weightless conditions, makes it possible to study the interaction between the cell and the gravitational field. In addition, the use of oocytes in this experiment is important from the point of view of the analysis of reproductive potential after the action of space flight factors and subsequent readaptation.

In this study, we analyzed the structure of the oocytes of the fruit fly *Drosophila melanogaster*, the maturation of which took place under space flight conditions. Oocytes began to be collected immediately after landing and continued for 12 h. The flies were then transferred onto fresh agar plates and oocyte collection continued for another 12 h. 

Based on the data in the literature [3,4,23] and our own studies [5,6], we expected to detect changes in the structure of the oocyte cytoskeleton and planned to evaluate its dynamics. However, few oocytes were obtained, especially in the first 12 h after landing, which made it possible to evaluate only one parameter. It is well known that the cytoskeleton determines the mechanical characteristics of cells, primarily cell stiffness [21,22,24,25,26,27], so this was measured by atomic force microscopy. The chosen indentation depth made it possible to estimate, first of all, the stiffness of the cortical cytoskeleton without introducing measurement artifacts into the obtained values.

The measurements performed showed that the stiffness of the cortical cytoskeleton of oocytes collected during the first 12 h was reduced compared to the corresponding control, which indicates a change in the structure of the cytoskeleton. Moreover, in oocytes collected in the next 12 h, the stiffness of the submembrane cytoskeleton remained below the control level, which indicates that the structure was not restored. Considering that the cortical cytoskeleton plays a key role in the formation of the contractile ring, it can be assumed that such changes in the structure of oocytes can disrupt cytokinesis in early embryos.

A decrease in cell stiffness is more often associated with the destruction of microfilaments [25,28], which is associated with a decrease in the content of actin and actin-binding proteins, including during the action of weightlessness and in the early period of readaptation [29,30,31]. During the second period of oocyte collection (between L + 12 h and L + 24 h), enough cells were obtained to allow both stiffness measurements and assessment of the content of various cytoskeletal proteins.

Indeed, the relative content of actin and some actin-binding proteins in the oocytes collected during the period L + 12 h—L + 24 h after landing was lower than in the control oocytes by about 30–50%. The relative content of microtubule components and intermediate filaments was reduced much more dramatically, by 70–80%. This comparison is more likely to support the view that cell stiffness is due to the structure of the microfilament network, since it does not decrease so dramatically. If the stiffness of oocytes was determined by microtubules, then a sharper decrease could be expected. However, such a proposal is speculative and requires a separate analysis. 

Of interest is the decrease in the content of actin-binding proteins involved in the formation of a developed network of microfilaments (alpha-actinin and spectrin alpha), but the content of the singed protein (fascin homologue), which binds microfilaments into dense bundles, remained at the control level. Taking into account the formation of oocytes under weightless conditions, the effect of overloads during the landing of the spacecraft and the period of adaptation to 1 g can be considered as an increase in external mechanical stress on the cells. The obtained results support our earlier proposed model of interaction between the cell and the gravitational field, in which an increase and decrease in external mechanical stress causes the deformation of the cortical cytoskeleton and the dissociation of various proteins from it [8,32].

## 5. Conclusions and Limitations of the Study

The results obtained indicate that the structure of the cortical cytoskeleton of *Drosophila melanogaster* oocytes, which matured during space flight, was compromised after landing and in the early period of adaptation to the Earth’s gravity, as evidenced by the cell stiffness and the content of cytoskeletal proteins. This pattern of oocyte structure can lead to problems with cytokinesis during the cleavage of early embryos.

The main limitation of this study is related to the small number of oocytes. A change in cell stiffness indicates a change in the structure of the cytoskeleton. However, to identify the main components that contribute to stiffness, it is required to determine the stiffness when using various inhibitors of cytoskeletal structures (cytochalasin, colchicine, etc.). The content of various cytoskeletal proteins, in the presence of a sufficient number of oocytes, should be supplemented by the visualization of their localization using confocal microscopy.

## Data Availability

All data generated or analyzed during this study are included in this article.

## References

[1] Ronca A.E., Baker E.S., Bavendam T.G., Beck K.D., Miller V.M., Tash J.S., Jenkins M. (2014). Effects of sex and gender on adaptations to space: Reproductive health. J. Women Health.

[2] Mishra B., Luderer U. (2019). Reproductive hazards of space travel in women and men. Nat. Rev. Endocrinol..

[3] Zhang S., Zheng D., Wu Y., Lin W., Chen Z., Meng L., Liu J., Zhou Y. (2016). Simulated Microgravity Using a Rotary Culture System Compromises the In Vitro Development of Mouse Preantral Follicles. PLoS ONE.

[4] Wu C., Guo X., Wang F., Li X., Tian X.C., Li L., Wu Z., Zhang S. (2011). Simulated microgravity compromises mouse oocyte maturation by disrupting meiotic spindle organization and inducing cytoplasmic blebbing. PLoS ONE.

[5] Ogneva I.V., Usik M.A. (2021). Mitochondrial Respiration in Drosophila Ovaries after a Full Cycle of Oogenesis under Simulated Microgravity. Curr. Issues Mol. Biol..

[6] Usik M.A., Golubkova M.A., Ogneva I.V. (2021). State of Drosophila melanogaster Ovaries after a Full Cycle of Gametogenesis under Microgravity Modeling: Cellular Respiration and the Content of Cytoskeletal Proteins. Int. J. Mol. Sci..

[7] Bartolák-Suki E., Imsirovic J., Nishibori Y., Krishnan R., Suki B. (2017). Regulation of Mitochondrial Structure and Dynamics by the Cytoskeleton and Mechanical Factors. Int. J. Mol. Sci..

[8] Ogneva I.V. (2022). Single Cell in a Gravity Field. Life.

[9] Schatten H., Lewis M.L., Chakrabarti A. (2001). Spaceflight and clinorotation cause cytoskeleton and mitochondria changes and increases in apoptosis in cultured cells. Acta Astronaut..

[10] Uva B.M., Masini M.A., Sturla M., Prato P., Passalacqua M., Giuliani M., Tagliafierro G., Strollo F. (2002). Clinorotation-induced weightlessness influences the cytoskeleton of glial cells in culture. Brain Res..

[11] Gaboyard S., Blanchard M.P., Travo C., Viso M., Sans A., Lehouelleur J. (2002). Weightlessness affects cytoskeleton of rat utricular hair cells during maturation in vitro. NeuroReport.

[12] Kacena M.A., Todd P., Landis W.J. (2003). Osteoblasts subjected to spaceflight and simulated space shuttle launch conditions. Vitr. Cell. Dev. Biol. Anim..

[13] Crawford-Young S.J. (2006). Effects of microgravity on cell cytoskeleton and embryogenesis. Int. J. Dev. Biol..

[14] Corydon T.J., Kopp S., Wehland M., Braun M., Schütte A., Mayer T., Hülsing T., Oltmann H., Schmitz B., Hemmersbach R. (2016). Alterations of the cytoskeleton in human cells in space proved by life-cell imaging. Sci. Rep..

[15] Thiel C.S., de Zélicourt D., Tauber S., Adrian A., Franz M., Simmet D.M., Schoppmann K., Hauschild S., Krammer S., Christen M. (2017). Rapid adaptation to microgravity in mammalian macrophage cells. Sci Rep..

[16] Thiel C.S., Tauber S., Seebacher C., Schropp M., Uhl R., Lauber B., Polzer J., Neelam S., Zhang Y., Ullrich O. (2019). Real-Time 3D High-Resolution Microscopy of Human Cells on the International Space Station. Int. J. Mol. Sci..

[17] Thiel C.S., Tauber S., Lauber B., Polzer J., Seebacher C., Uhl R., Neelam S., Zhang Y., Levine H., Ullrich O. (2019). Rapid Morphological and Cytoskeletal Response to Microgravity in Human Primary Macrophages. Int J Mol Sci..

[18] Ogneva I.V., Belyakin S.N., Sarantseva S.V. (2016). The Development of Drosophila Melanogaster under Different Duration Space Flight and Subsequent Adaptation to Earth Gravity. PLoS ONE.

[19] Ogneva I.V., Zhdankina Y.S., Kotov O.V. (2022). Sperm of Fruit Fly Drosophila melanogaster under Space Flight. Int. J. Mol. Sci..

[20] Tran S.L., Welte M.A. (2010). In-vivo centrifugation of Drosophila embryos. J. Vis. Exp..

[21] Ogneva I.V. (2010). Transversal stiffness of fibers and desmin content in leg muscles of rats under gravitational unloading of various durations. J. Appl. Physiol..

[22] Udartseva O.O., Zhidkova O.V., Ezdakova M.I., Ogneva I.V., Andreeva E.R., Buravkova L.B., Gollnick S.O. (2019). Low-dose photodynamic therapy promotes angiogenic potential and increases immunogenicity of human mesenchymal stromal cells. J. Photochem. Photobiol. Biol..

[23] Zhang S., Wu Y., Weng Y., Xu Z., Chen W., Zheng D., Lin W., Liu J., Zhou Y. (2017). In Vitro Growth of Mouse Preantral Follicles under Simulated Microgravity. J. Vis. Exp..

[24] Mathur A.B., Collinsworth A.M., Reichert W.M., Kraus W.E., Truskey G.A. (2001). Endothelial, cardiac muscle and skeletal muscle exhibit different viscous and elastic properties as determined by atomic force microscopy. J. Biomech..

[25] Costa K.D. (2006). Imaging and probing cell mechanical properties with the atomic force microscope. Methods Mol. Biol..

[26] Cai X., Gao S., Cai J., Wu Y., Deng H. (2009). Artesunate induced morphological and mechanical changes of Jurkat cell studied by AFM. Scanning.

[27] Ogneva I.V., Buravkov S.V., Shubenkov A.N., Buravkova L.B. (2014). Mechanical characteristics of mesenchymal stem cells under impact of silica-based nanoparticles. Nanoscale Res. Lett..

[28] Collinsworth A.M., Zhang S., Kraus W.E., Truskey G.A. (2002). Apparent elastic modulus and hysteresis of skeletal muscle cells throughout differentiation. Am. J. Physiol. Cell Physiol..

[29] Ogneva I.V., Maximova M.V., Larina I.M. (2014). Structure of cortical cytoskeleton in fibers of mouse muscle cells after being exposed to a 30-day space flight on board the BION-M1 biosatellite. J. Appl. Physiol..

[30] Ogneva I.V., Biryukov N.S., Leinsoo T.A., Larina I.M. (2014). Possible role of non-muscle alpha-actinins in muscle cell mechanosensitivity. PLoS ONE.

[31] Ogneva I.V., Gnyubkin V., Laroche N., Maximova M.V., Larina I.M., Vico L. (2015). Structure of the cortical cytoskeleton in fibers of postural muscles and cardiomyocytes of mice after 30-day 2-g centrifugation. J. Appl. Physiol..

[32] Ogneva I.V. (2013). Cell mechanosensitivity: Mechanical properties and interaction with gravitational field. BioMed Res. Int..

